# Spectro-Electrochemical Properties of A New Non-Enzymatic Modified Working Electrode Used for Histamine Assessment in the Diagnosis of Food Poisoning

**DOI:** 10.3390/foods12152908

**Published:** 2023-07-31

**Authors:** Stefan-Marian Iordache, Ana-Maria Iordache, Alexei Zubarev, Stefan Caramizoiu, Cristiana Eugenia Ana Grigorescu, Silviu Marinescu, Carmen Giuglea

**Affiliations:** 1 Optospintronics Department, National Institute for Research and Development for Optoelectronics—INOE 2000, Atomistilor 409, 077125 Magurele, Romania; stefan.iordache@inoe.ro (S.-M.I.); krisis812@yahoo.co.uk (C.E.A.G.); 2 National Institute for Laser, Plasma and Radiation Physics, 077125 Magurele, Romania; 3 National Institute for R&D in Microtechnologies IMT-Bucharest, 126A Erou Iancu Nicolae Str., 077190 Voluntari, Romania; stefancaramizoiu@yahoo.com; 4 Department of Plastic Surgery, University of Medicine and Pharmacy “Carol Davila”, Eroii Sanitari Bvd., No. 8, Sector 5, 050471 Bucharest, Romaniagiugleacarmen@yahoo.com (C.G.)

**Keywords:** histamine, non-enzymatic sensor, cyclic voltammetry, Raman spectroscopy, electropolymerization, food poisoning

## Abstract

We successfully prepared a non-enzymatic sensor based on a graphene-thiophene composite for histamine detection. The self-assembling properties of the thiophene onto Au support and the high electrical conductivity of graphene encouraged the choice of this type of composite. The composite was deposited via electrochemical polymerization onto the Au layer of a screen-printed microelectrode. The electropolymerization and electrochemical detection of histamine were both achieved by cyclic voltammetry. Two types of electrolytes were used for the electrochemical detection: (a) phosphate buffer solution (PBS), which showed low-intensity redox peaks for histamine; and (b) trichloroacetic acid (TCA) 0.01 M, which showed improved results over PBS and did not damage the microelectrode. For the concentration range of 100–200 mg/kg, the sensor shows a linear regression pattern for the oxidation peak fitted on the equation *I_pa_* = 123.412 + 0.49933 × *x*, with *R*^2^ = 0.94178. The lowest limit of detection was calculated to be 13.8 mg/kg and the limit of quantification was calculated at 46 mg/kg. These results are important since by monitoring the amount of histamine in a food product, early onset of spoilage can be easily detected, thus reducing foodborne poisoning and food waste (by recycling products that are still edible).

## 1. Introduction

“Food poisoning” is defined by both the National Health Service (NHS), UK, and the Center for Disease Control (CDC), USA, as an illness produced by eating contaminated food. *Salmonella, Campylobacter, Escherichia coli, Listeria, Vibrio, Staphylococcus aureus,* and *Clostridium perfringens* count among the most frequently encountered pathogens [1,2]. Bacterial development in food releases an important amount of histamine, which is responsible for scombroid poisoning and allergy responses in humans [3]. Although scombroid poisoning is in the top three most common agents causing foodborne disease, its action mechanism is still unclear [4]. Usually, histamine is inactivated in the intestines and should not produce illness. However, the high concentration of histamine in urine samples collected from patients admitted with food poisoning, associated with the well-known histamine heat stability (e.g., cooking does not neutralize it), proves otherwise [5]. The symptoms are mild and can easily be mistaken for other gastroenterological problems. The diagnosis is given by the presence of a definitive mark, that is the sunburn-like rash on the face and neck areas. The diagnosis can also be guided by the anamneses, most patients remember being sick after eating fish [6,7]. Left untreated, the intoxication subsides within 24 h. In severe cases, respiratory distress and vasodilatory shock could develop, which in turn could lead to complications [8].

Thus, it is important to rapidly identify the level of histamine and correlate it with the level of bacterial contamination in food products. Extraction of histamine from the complex food matrix has been classically performed via solid phase/liquid–liquid extraction [9,10,11,12] or solid/liquid phase microextraction [13,14] to remove the interferents and the impurities. But the extraction step presents several disadvantages such as tedious steps, large sample and organic solvent volumes, low selectivity and reproducibility, and environmental pollution [9]. The most popular methods for detection and quantification of histamine, following extraction, are high-performance liquid chromatography (HPLC) [15], liquid chromatography coupled with mass spectrometry (LC-MS) [16], absorbance [17], and fluorometry [18,19]. Additionally, surface-enhanced Raman spectroscopy (SERS) [20,21,22], and ELISA [23,24], have also been intensely researched as alternative routes for histamine detection. These detection methods are based on the activity of enzymes and nanoenzymes, and their bioconjugation of histamine to produce an optoelectronic signal [25]. However, optoelectronic transducers exhibit a few drawbacks, such as the requirement of special equipment, possible influence from natural radiation, whose removal would involve a compensation method, and the response being delayed because of the mass transfer. Also, miniaturization is difficult to attain.

Since the European Union has a strict limit of 100 mg∙kg^−1^ (or 100 ppm since 1 mg∙kg^−1^ = 1 ppm)) for histamine concentration in food products, and the World Health Organization established a maximum safe level of 200 mg∙kg^−1^ (200 ppm) [26], alternatives to the expensive, highly specialized equipment necessary for the identification and quantification of histamine have been pursued. Electrochemical sensors are a great alternative. Compared to the chromatography, fluorescence, and spectrophotometry techniques that require derivatization [27], electrochemical sensors can provide an on-site evaluation of the samples.

The aim of an electrochemical investigation is to establish a direct connection between the current response and the target analyte (histamine), which is achieved by recording the oxidation and the reduction processes during a redox reaction [28]. Several electrochemical sensors have been explored. Li et al. [3] studied a sensitive layer based on *p*-aminobenzene sulfonic acid and Au-nanoparticles electropolymerized onto a glassy carbon electrode. The sensor exhibited a low detection limit, but the recovery percentage was about 87–89%, depending on the alcohol type, and requiring an incubation period in the histamine-containing sample. Sahudin et al. [29] prepared an impedimetric sensor based on a hydroxyl functionalized Schiff base zinc (II) complex with titanium dioxide nanoparticles, with a better recovery of 94–97%. For the impedimetric measurements, they used a solution containing a 0.1 M PBS buffer and 5 mM of K_3_[Fe(CN)_6_]/K_4_[Fe(CN)_6_]. Other materials used for the detection of histamine were metal-organic framework [30], graphene [31], ZnO/TiO_2_ [32], and CeO_2_–PANI [33].

DNA was also used for the evaluation of histamine in the food sample. Xu et al. [34] used an Apt/AuNFs/ITO sensor in combination with Au@FeCo nanohybrids to act as an electrochemical mediator in 3,3′,5,5′-tetramethylbenzidine (TMB)-H_2_O_2_ electrolyte system. Even though the sensor exhibited a wide detection range (1–5000 nmol∙L^−1^), the fabrication method had been requiring many steps and complicated procedures. Other authors used biological enzymes such as diamine oxidase immobilized in phase-change microcapsules (composed of an n-docosane core, a TiO_2_ shell, and an electroactive polyaniline/ZnO composite coating layer), and assessed the quantity of histamine via in situ thermo-temperature regulation [35]. This type of sensor uses heat energy storage, and latent heat release, to electrochemically evaluate the levels of histamine in food samples. Zhang et al. developed an even more spectacular technique: fluorescamine-derivatized, thin-layer chromatography surface-enhanced Raman spectroscopy for evaluating histamine [36]. This method allows for the visualization of histamine and a detection limit of 9 ppb. Graphene-based materials have been used as sensors for the electrochemical detection of histamine [31,37,38,39] because they combined the chemical stability, high electrical, and thermal conductivity of the graphene with the excellent catalytic activity and sensitivity properties of the biomolecule/polymer. These hybrid materials proved excellent as sensors because they avoided agglomeration of the nanoparticles and expressed an increase in the number of active sites.

A highly sensitive sensor for histamine, based on horseradish peroxidase, was developed by Yang et al. and involved several steps [40]: (1) first antibodies are immobilized onto the support molecule (e.g., graphene, MWCNT, etc.); (2) then, histamine and/or histamine-tagged molecules (defined as template molecules) are incubated with the antibodies and support; (3) the “histamine/histamine-derivative + antibodies-support” are deposited onto the glassy carbon electrode (GCE) via direct deposition or polymerization of 3,3′-dimethoxybenzidine (DB) in the presence of H_2_O_2_; (4) the template molecules (e.g., histamine/histamine derivative) are removed from the sensitive deposited/electropolymerized film to create the selective active site for the analyte; (5) the sensitive film is incubated in the sample so that the analyte (histamine/histamine derivative) can occupy the vacant active site; and (6) the variation of the electrochemical current of the electrode in Fe(CN)_6_^3−/4−^ solution vs. the concentration of histamine is recorded. A simplified version of this process is presented in ref. [41].

As an alternative to all these complicated procedures and mediator solutions, we propose a novel electrochemical sensitive layer based on graphene and thiophene for the detection of histamine in the 100–200 mg∙kg^−1^ interval. We identified some advantages of this new structure: (1) thiophene was selected as a semiconductive polymer due to its ability to form self-assembled monolayers on Au surfaces; (2) the chemically functionalized surface of the Au screen-printed electrodes work in small volume solutions (50 µL); and (3) derivatization of histamine is performed using trichloroacetic acid (TCA), which also acts as a mediator. TCA is less expensive than Au colloids, free sialic acid, or horseradish peroxidase, and is chemically stable for long periods of time.

## 2. Materials and Methods

### 2.1. Reagents and Equipment

Thiophene (98%, Sigma Aldrich, Darmstadt, Germany), trichloroacetic acid (99%, Sigma Aldrich, Darmstadt, Germany), histamine dihydrochloride (99%, Alfa Aesar, Ward Hill, MA, USA), L-adrenaline (99%, Alfa Aesar, Ward Hill, MA, USA.), and serotonin (99%, Alfa Aesar, Ward Hill, MA, USA) were used as received without purification. Phosphate-buffered saline solutions with a pH = 7.4 and graphene nanoplates were acquired from Sigma Aldrich. Tween 20^®^ was purchased from ThermoFisher (Kandel, Germany). DRP-220AT microelectrodes (Au-based working electrode, with an area of 0.125 cm^−2^) from DropSens (Metrohm DropSens S.L., Llanera, Spain) were used as support for the deposition of graphene-thiophene composite.

Raman spectroscopy was performed with a LABRAM HR 800 spectrometer, with 633 nm excitation laser wavelength, 2.11 mW laser power at the sample surface, 0.5 cm^−1^ resolution, 10 s accumulation time, for each measurement. Electrochemical characterization was performed with an OrigaFlex 0.5A modular electrochemical system from OrigaLys ElectroChem SAS (Rillieux-la-Pape, Lyon, France), a three-electrode cell, and a module for microelectrodes.

### 2.2. Composite Solution Preparation

A quantity of 1.03 mg graphene nanoplates and 500 µL thiophene were dispersed in a 51 mL phosphate buffer solution (PBS) and 2 µL of Tween 20 with a Hielscher ultrasound probe (Hielscher Ultrasonics, Teltow, Germany) and centrifuged at 4000 rpm for 10 min to separate the deposit from the supernatant. The supernatant was collected and used in the subsequent electrochemical polymerization. The final pH of the solution was 6.6.

### 2.3. Electrochemical Polymerization of Graphene-Thiophene Composite

DRP-220 AT microelectrodes have a three-electrode configuration: the working electrode, and the auxiliary one, are made of Au, and the reference electrode is made of Ag. The electrode surface was washed and dried before experiments. Parafilm was then wrapped around the sensor only covering the reference and auxiliary electrodes, with the working electrode left bare. In total, 50 mL of supernatants were placed in the electrochemical cell, together with the reference electrode (saturated calomel) and the auxiliary electrode (Pt wire). Cyclic voltammetry was initiated with a duration of 20 cycles. The potential was swept between −1000 mV and 1000 mV with a scan rate of 100 mV∙s^−1^. The sensors were washed in deionized water and dried with an IR lamp before use. Three identical sensors were fabricated to be tested in different histamine solutions.

### 2.4. Preparation of Histamine Solutions

Two solutions were prepared: (1) a stock solution of histamine in PBS, 263 mg∙kg^−1^; and (2) a stock solution of histamine in 0.01M TCA, 1253 mg∙kg^−1^. Lower concentrations of histamine (1–200 mg∙kg^−1^) were achieved using successive dilutions of the stock solutions. The appropriate TCA concentration was determined by preparing 5 solutions (0.01 M, 0.02 M, 0.05 M, 0.1 M, and 0.5 M) and by plotting the anodic peak intensity, and charge, vs. TCA concentration. The lowest value of the two properties corresponds to the optimal TCA concentration (0.01 M). In total, 50 µL of each histamine solution was placed onto the microelectrode and cyclic voltammetry was performed.

### 2.5. Preparation of the Interfering Solution

A solution containing 178.2 mg∙kg^−1^ L-adrenaline, 249.8 mg∙kg^−1^ serotonin, and 673.6 mg∙kg^−1^ histamine was prepared by dissolving the appropriate amounts of analytes (8.91 mg L-adrenaline, 12.49 mg serotonin, and 33.68 mg histamine) in 50 mL of 0.01 M TCA. A volume of 50 µL was deposited onto the microelectrode and the electrochemical response was recorded.

### 2.6. Electrochemical Characterization

Cyclic voltammetry was carried out with the module for microelectrodes of the OrigaFlex potentiostat in the intervals [−500; 1000] mV, [−750; 1000] mV, with a 200, 100, 50, and 25 mV∙s^−1^ scanning rate, 1:7 sampling rate, no ohmic drop component, and 5 cycles. The current density is expressed as µA∙cm^−2^ by dividing the current intensity by the geometric area of the electrode.

## 3. Results and Discussions

### 3.1. Electropolymerization of the Graphene-Thiophene Sensitive Layer

Figure S1 shows the spectrum of the electrochemical polymerization of the graphene-thiophene composite. In total, 20 cycles of electropolymerization were performed. The shape and the height of the response is determined using the redox processes involved at the electrode/electrolyte interface [28]. The voltammogram shows that the graphene-thiophene composite has a single oxidation peak, at 58.4 mV, and two reduction peaks, at ≈98 mV, and 424 mV. The oxidation peak is well-defined and sharp, being assigned to the oxidation of the composite. Its shape is produced using the two conductive components: (a) the highly conductive graphene, which efficiently promotes the diffusion of the thiophene monomer on the surface of the electrode; and (b) thiophene is oxidized and self-assembles in a regular structure on the surface of the Au electrode, forming polythiophene and cementing the graphene in a sandwich-like structure.

The first reduction peak, at 98 mV, is accompanied by a shoulder that diminishes with the increase of the number of cycles until vanishing from cycle No. 16 onward. This peak is produced by the reduction of the polythiophene film (deprotonation phase [42]). The second reduction peak has a broad shape owing to a capacitive component, a graphene-thiophene composite most likely. Since graphene is already reduced and can store a limited amount of charge, reduction can only occur in the polymer film.

It is worth noticing that the potentials of oxidation are slightly shifted to smaller values, e.g., the first cycles have a potential value of 72 mV and slowly decrease, becoming stable at 58.4 mV for 13 cycles. The reduction peaks have different behavior: the first reduction peak starts at −26 mV and becomes stable at around 96.4–98 mV, then begins a slow increase, becoming stable at 101.6 mV in the last three cycles. The second reduction peak begins at 220.6 mV and becomes stable at 424 mV after cycle 13. These variations in potentials settle after cycle No. 17. Since the variation in potentials is accompanied by an increase in the current density, we can assume that the polymeric film “grows” on the surface of the electrode and increases the electrical resistance, thus leading to an increase/decrease of the potential values [43,44,45].

**Figure 1:**
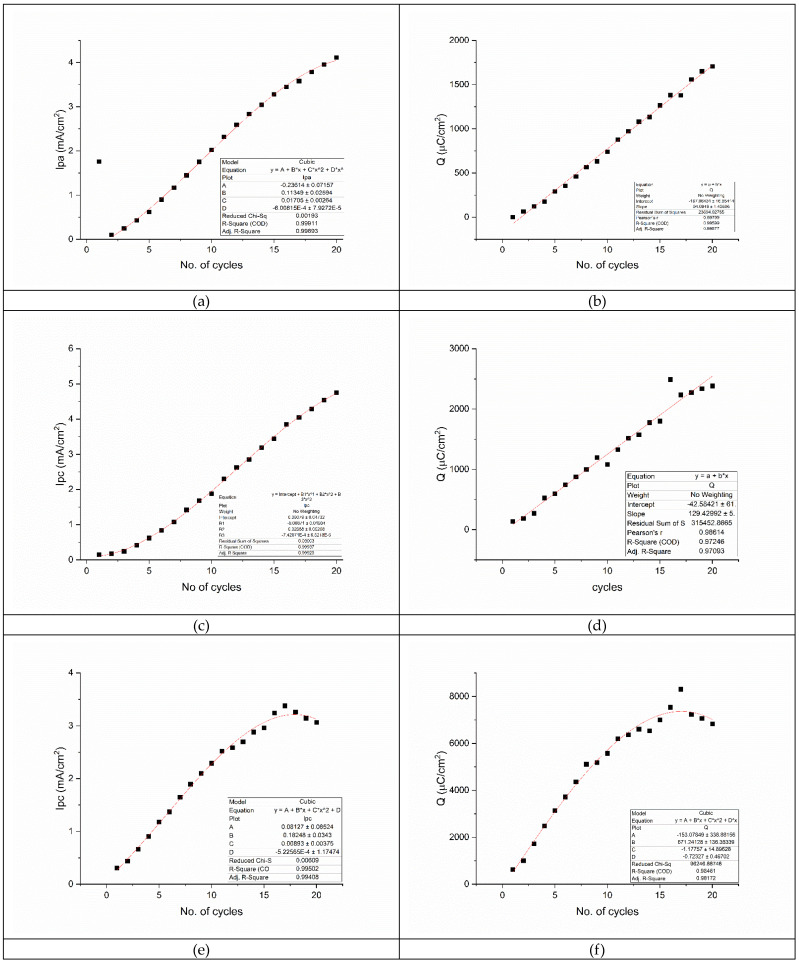
Variation of the peak intensities and charges of the polymerization process vs. the No. of cycles: (**a**) anodic peak intensity vs. No. of cycles; (**b**) charge variation for the anodic peak vs. No. of cycles; (**c**) first cathodic peak (−98 mV) intensity vs. No. of cycles; (**d**) charge variation for the first cathodic peak (−98 mV) vs. No. of cycles; (**e**) second cathodic peak (424 mV) intensity vs. No. of cycles; and (**f**) charge variation for the second cathodic peak (424 mV) vs. No. of cycles.

Analyzing each parameter of the voltammogram (peak intensity and charge) as a function of the number of cycles, one can observe that the oxidation and first reduction peak of the data are similar. By calculating the *Ipa/Ipc1* ratio, where *Ipa* is the current density of the oxidation peak (58.4 mV) and *Ipc1* is the current density of the first reduction peak (98 mV), we obtain values between 1.07 and 0.86, which indicates a reversible reaction (the same number of electrons are necessary for oxidation and reduction of the polymer. These results are compiled in Figure 1. The *Ipa* (Figure 1a) and *Ipc1* (Figure 1c) can be fitted on a 3rd degree polynomial function, giving similar equations, and close *R*^2^ values:

(1) $$Ipa=-0.23614+0.11349\times x+0.01705\times x^{2}-6\cdot 10^{-4}\times x^{3}$$

*R*^2^ = 0.99911

(2) $$Ipc=0.09079-0.00871\times x+0.02688\times x^{2}-7.42\cdot 10^{-4}\times x^{3}$$

*R*^2^ = 0.99937

Other studies [43,45], showed that this behavior is produced by the growth of the polythiophene film, which reduces the diffusion of the monomer to the surface of the electrode and hinders its oxidation by dropping the surface potential across. Generally, after the 20th cycle, the growth of the polymeric film reaches a saturation point, the polythiophene forms a membrane that envelops the working electrode, and the potential and current density settle in a fixed position. Even though we additionally performed 40 cycles, we did not reach saturation of the electrode for this composite. This phenomenon is evident from the graph of charge vs. No. of cycles (Figure 1b,d), which follows a linear equation for both oxidation and reduction parts:

(3) $$Q\left(oxidation\right)=-167.06431+94.0946\times x$$

*R*^2^ = 0.99599

(4) $$Q\left(reduction\right)=-42.58421+129.42992\times x$$

*R*^2^ = 0.97246

This behavior does not hinder our results. We focused on the electrodeposition of a thin layer of composite on the surface of the Au support, i.e., the smallest amount of composite, to ensure enough active sites to bind with the derivatized histamine.

From Equations (3) and (4), we can assume that the current density (intensity of the oxidation and reduction peaks) will reach a saturation point after more than 40 cycles, but the charge exchange between the working electrode and composite will continue without reaching saturation. This behavior is explained by the high conductivity of both graphene and polythiophene, which together form a highly charged composite.

For the second reduction peak, the broad plateau at 424 mV, both current density (intensity of the reduction peak, Figure 1e,f), follow a cubic polynomial equation:

(5) $$Ipc=0.08127+0.18248\times x+0.00893\times x^{2}-5.22\cdot 10^{-4}\times x^{3}$$

*R*^2^ = 0.99502

(6) $$Q\;\left(reduction\right)=-153.07849+671.24128\times x-1.17757\times x^{2}-0.72327\times x^{3}$$

*R*^2^ = 0.98461

Since both current density and charge follow a higher polynomial order, this means that the reduction peak will reach a saturation point followed by a decrease. This point has been reached for this reduction peak at cycle No. 17, which has the highest current density and charge, and is followed by a decrease in both parameters for the subsequent cycles.

By comparing the charge from the oxidation and reduction peaks, it is obvious that the charge exchanged in the film oxidation is lower than the one consumed during reduction. This could be related to the differences in film thickness, but, most likely, the graphene is responsible for increasing the electroactivity of the composite and acting as a “crosspolymer”, enhancing the capacitive potential.

### 3.2. Raman Spectroscopy of the Sensitive Layer

Figure S2 shows the Raman spectra for the graphene, thiophene, graphene-thiophene composite, and the clean electrode surface. The graphene spectrum presents the two representative peaks (the D and G bands corresponding to the 1350 cm^−1^ and 1588 cm^−1^ peaks) assigned to the scattering of the incident light on local defects/disorders and the Raman-active phonon mode of the C−C bonds in the graphitic structure [46].

The Raman spectrum of the thiophene shows the peaks at 461 cm^−1^ (C-S stretching mode), 615 cm^−1^ (π conjugated ring), 755 cm^−1^ (asymmetric distortion of the ring), 837 cm^−1^ (C-H stretching mode), 1040 cm^−1^ and 1088 cm^−1^ (in plane/out of plane ˃ CH_2_ stretching vibration), 1369 cm^−1^ (C-C bond in the ring), and finally 1412 cm^−1^ (rocking mode of the C-H group) [47,48,49].

For the polymer, there are some important peaks relevant to determine the polymerization ratio. Those peaks are at 461 cm^−1^ and 837 cm^−1^ and are assigned to the C-S-C bond and C-H stretching mode, respectively. We presume that when polymerization occurs, the vibration of the C-S bond increases (the two thiophene molecules are “connected” in a *trans* position) as compared to the C-H stretching mode, which dominates in the free monomer. We calculated a polymerization ratio of 5.4% for the monomer, expressed as:

$$R_{polymerization}=\frac{I_{461cm-1}}{I_{837cm-1}}$$

**Figure 2:**
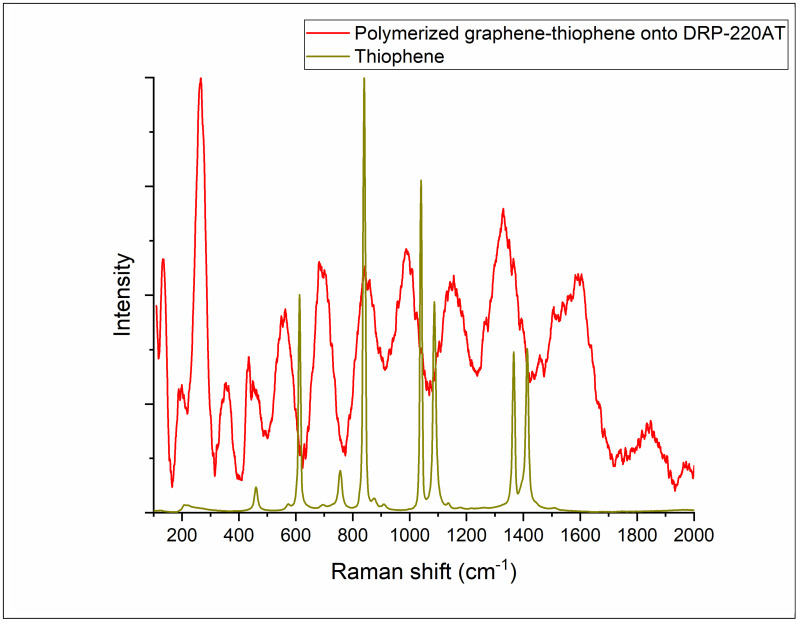
Raman spectra of the thiophene and of the electropolymerized film deposited onto the Au working electrode.

This value is not surprising since it is well known that all organic monomer solutions have polymeric impurities once monomers undergo polymerization under natural light. That is why distillation of the monomer solution before use is recommended. Using the same formula, we found a polymerization ratio of 61.95% for the polymeric film deposited onto the Au working electrode. For better visualization, we overlaid the thiophene spectrum with the spectrum of the polymerized composite on the DRP-220AT microelectrode (Figure 2).

This polymerization ratio for the graphene-thiophene composite is consistent with the number of cycles; we chose only 20 cycles of electropolymerization in order to deposit a thin layer of composite on the Au microelectrode.

### 3.3. Detection of Histamine in Phosphate Buffer Solution (PBS)

Further, the sensor was tested for different concentrations of histamine. We chose a phosphate buffer solution with a pH of 7.4 as the electrolyte solution. From the stock solution containing 263 mg∙kg^−1^ of histamine, we prepared, via successive dilution, another eight concentrations ranging from 2 to 200 mg∙kg^−1^. Figure S3 shows the cyclic voltammetry (CV) of the solutions on the new graphene-thiophene composite electrode. The voltammograms have no double charge layer, which means that diffusion of histamine on the surface of the electrode happens very fast and shows a reversible reaction.

There are only two peaks: an oxidation peak and a reduction one. At first glance, the CVs seem to have a uniform decrease in current density (i.e., the intensity of the peaks) with the concentration of histamine. Still, the graphical representations of the current density vs. histamine and charge vs. histamine concentration, (Figure S4b) show a non-linear response of the two variables. When calculating the ratio of *I_pa_/I_pc_*, we found that it equals unity (R ≅ 1). This means that the number of electrons involved in oxidation is the same as the number of electrons released during reduction. In Figure S4b, the charge exchanged during reduction reaches a maximum for a value of 10 mg∙kg^−1^ histamine, but decreases drastically for the subsequent concentrations, allowing the oxidation charge to increase for 100 mg∙kg^−1^ and 150 mg∙kg^−1^ histamine.

This shows that although very sensible to histamine, the sensor gives an uneven response to different concentrations of the analyte. Moreover, when inspecting the sensor, we observed that the counter electrode and the reference electrode were completely black. The reaction of the sensor materials with the PBS is responsible for the un-uniform response to histamine and the very small double-charge layer. We conclude that PBS is not an appropriate electrolyte for the identification of histamine.

### 3.4. Determination of the Appropriate TCA Concentration

The second electrolyte to be tested was trichloroacetic acid. We know from previous experiments [50,51] that a concentration of TCA above 0.5 M will react with the plastic isolation of the DRP-220AT (the plastic will swell and exfoliate, revealing the contacts of the electrodes). So, for our tests, we employed five concentrations of TCA ranging from 0.01 to 0.5 M. The results are shown in Figure S5. In the CVs (Figure S5a), TCA exhibits low-intensity oxidation peaks (two are more pronounced, around 0 mV and 700 mV) and one broad low-intensity reduction peak (around −250 mV). By plotting the results of the CVs in Figure S5b, we can see that the lowest intensities for both current densities and charge are for the lowest concentration of TCA. This is important since the current density and charge can interfere when detecting histamine in the histamine-TCA system. Also, no reaction with the counter electrode, or reference electrode, was seen. To conclude, we chose the 0.01 M TCA as an electrolyte for the dispersion of histamine.

### 3.5. Evaluation of Histamine in 0.01 M TCA

**Figure 3:**
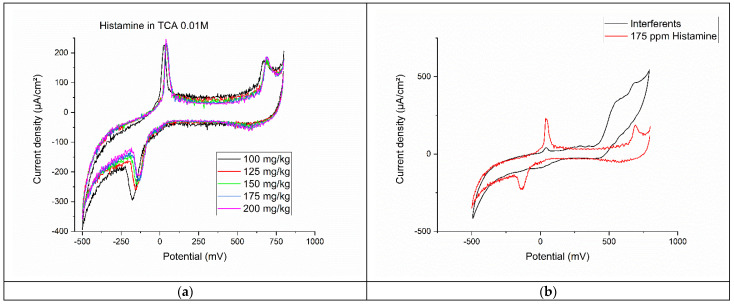
(**a**) Cyclic voltammetry of different concentrations of histamine in 0.01M TCA. (**b**) Electrochemical analysis of a solution containing histamine and two interferents.

Five solutions of histamine in 0.01 M of TCA were prepared: 100 mg∙kg^−1^, 125 mg∙kg^−1^, 150 mg∙kg^−1^, 175 mg∙kg^−1^, and 200 mg∙kg^−1^. Those concentrations are representative of the level of histamine commonly found in foods that are at their end of the shelf life. The response of the graphene-thiophene composite sensor to histamine-TCA is shown in Figure 3a. The voltammograms indicate two oxidation peaks, at ≈41 mV and ≈687 mV, and one reduction peak at ≈173 mV. The presence of the double charge layer demonstrates the presence of a diffusion layer near the surface of the modified electrode, responsible for the movement of the histamine-TCA complex from high-concentration to low-concentration areas. From the cyclic voltammetry, the intensity of the reduction peak seems to be proportional to the concentrations.

**Figure 4:**
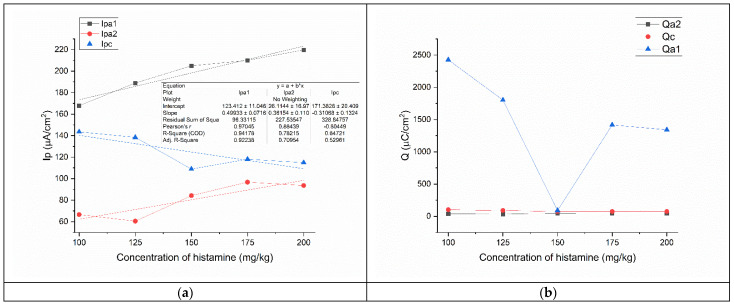
Variation of the concentration of histamine in TCA with (**a**) peak intensities and (**b**) charge.

By plotting the parameters of the CVs vs. the concentrations of histamine (Figure 4a) and by linearly fitting the experimental points, we obtain three linear equations for each parameter:

(7) $$I_{pa1}=123.412+0.49933\times x$$

*R*^2^ = 0.94178

(8) $$I_{pa2}=26.1144+0.36154\times x$$

*R*^2^ = 0.78215

(9) $$I_{pc}=171.3828-0.31068\times x$$

*R*^2^ = 0.64721

The first oxidation potential has the highest *R*^2^, which means it is the best fitting for a linear dependence with the concentration of histamine. For the Ipc, the *R*^2^ is much lower, at 0.64721. The detection limit (LOD) was calculated to be 13.8 mg∙kg^−1^ (3S/N), while the limit of quantification (LOQ) was calculated to be 46 mg∙kg^−1^. The highest calculated concentration of histamine that can be detected is 551 mg∙kg^−1^. TCA is used for the derivatization of histamine; thus, it forms a chemical complex that is electrochemically active. Its oxidation potential is at ≈41 mV. The increase in histamine concentration produces an increase in the number of histamine-TCA complexes and determines an increase in the intensity of the oxidation peak [50].

The charge graph vs. concentration of histamine (Figure 4b) shows what seems a linear dependency of the anodic charge from oxidation peak 2 and the reduction peak, and a non-linear dependency of the anodic charge of anodic peak 1. However, when fitting the graphs, we observe that all charge vs. histamine concentration variations are non-linear, with an *R*^2^ < 0.5.

Further on, the square root of the scanning speed was plotted against the current density of the 200 mg∙kg^−1^ histamine sample (Figure S6). The linear plot was given by the equation:

(10) $$I_{pc}=-103.05969+3.72311\times x$$

*R*^2^ = 0.99893

This almost perfect linear dependency establishes the diffusion of the histamine-TCA complex as the rate-determining step. This is especially true since we suppressed the two other mass transport modes: electromigration, by adding an excess of acidic solution (compared to the analyte concentration) and convection, by using a low-volume stationary solution.

### 3.6. Analysis of Interferents

The graphene-thiophene sensor was tested on L-adrenaline and serotonin as interferents for histamine. In total, 50 µL of a solution containing 178 mg∙kg^−1^ of L-adrenaline, 249 mg∙kg^−1^ of serotonin, and 673 mg∙kg^−1^ of histamine was tested on the modified electrode. We chose a higher concentration of histamine compared with the two interferents because we focused on the food products found at the end of the shelf life (thus spoiled or contaminated), which have a high amount of histamine. Another factor in choosing a 600 mg∙kg^−1^ concentration of histamine is a higher maximum admitted limit in other countries, e.g., U.S.A. and P.R.C. that have a 500 mg∙kg^−1^ limit of histamine in fish products [26].

The values we obtained from the experimental data were: oxidation potential = 41.8 mV, anodic peak intensity = 29.839 µA∙cm^−2^, reduction potential = 12.5 mV, and cathodic peak intensity = 16.622 µA∙cm^−2^. Figure 3b shows the results of the interference solution vs. the 175 mg∙kg^−1^ histamine solution. By calculating the concentration of histamine from the interferents’ solution using Equation (7), we obtain a value of 738 mg∙kg^−1^ for histamine. This value is slightly higher than the 673 mg∙kg^−1^ we used for the interferent solution, giving a signal recovery [29] of 109%.

(11) $$recovery=\frac{determined\;concentration}{known\;concentration}\times 100$$

This is because the interferent solution contains a histamine concentration well above the maximum detection limit. It is important to notice that the sensor does not misidentify a spoiled food product as a fresh one (no false positive results).

Table 1 summarizes the most important analytical parameters of our sensor (the formulas for their calculation are given in the Supplementary files). By comparing our results with those obtained by other authors (Table 2) we can see that our sensor exhibits a moderate sensitivity. Some of the most important traits, in our opinion, are that this sensor shows a LOD of 13.8 mg∙kg^−1^ and a good recovery even for concentrations of histamine well beyond its maximum limit.

The caution level of histamine in food samples, particularly in fish because scombroid poisoning appears after ingestion of seafood, has been debated for years. Levels of 20 mg∙100 g^−1^ of product, or 50 mg∙100 g^−1^, were suggested by the US Food and Drug Administration [52]. At the same time, the CDC published a report stating that levels as high as 20 mg/100 g of product are sufficient to induce poisoning in humans [53]. In Europe, the maximum admissible level of histamine in fish is 200 mg∙kg^−1^ (for fresh fish) and 400 mg∙kg^−1^ (for fish in brine) [54]. Older reports suggested that above 100 mg∙100 g^−1^ of fish product is toxic for consumption [55]. Other recent reports sustained this point of view [4,6,56] by identifying the pathogens that produce histamine in large amounts and quantifying the levels of histamine in fish products (ranging from 50 to 250 mg∙100 g^−1^). Since scombroid poisoning is not life threatening, the maximum levels could be set at higher values. Some reasons could be that all companies introduce some sort of preservative and antimicrobial-control substances that inhibits the bacteria which produce histamine in the first place.

## 4. Conclusions

We fabricated a novel graphene-thiophene sensor based on the electropolymerization of the polymer on the Au working electrode of a DRP-220AT microelectrode support. The polymerization ratio was 61.95% as calculated from the Raman spectrum. This sensor was tested on different solutions of histamine in PBS and TCA. The solution of histamine in PBS was inappropriate because it reacted with both the auxiliary and the reference electrodes. Therefore, the TCA solution was selected as an alternative. However, the TCA solution has been of low concentration too, because a higher concentration (>0.5 M TCA) will swell and exfoliate the plastic isolation of the electrode support. We selected 0.01 M of TCA and tested the sensor to a range of histamine concentrations between 100 and 200 mg∙kg^−1^. The variation of the first anodic peak intensity vs. histamine showed the best fit, with an *R*^2^ = 0.941478 which indicated a recovery value of 109% for a concentration of histamine exceeding the maximum detection limit. The sensor responds well to concentrations of histamine in the linear range from 13.8 to 551 mg∙kg^−1^, which corresponds to fresh and spoiled food products, respectively, indicating that the 0.01M TCA is an appropriate derivatization agent for histamine.

For other perspectives, we intend to increase the number of polymerization cycles and decrease the concentration of histamine <100 mg∙kg^−1^, to test the sensor in real-life samples of meat, and to corroborate the electrochemical results with HPLC/GS-MS studies. Since foodborne diseases have a major impact on health, this type of sensor is a desirable device to be introduced in the healthcare practice.

## Figures and Tables

**Table 1:** Summary of the most important analytical parameters of our histamine detection system.

Material	Supporting Electrolyte	Concentration Range (mg∙kg^−1^)	LOD (mg∙kg^−1^)	LOQ (mg∙kg^−1^)	Maximum Limit of Detection (mg∙kg^−1^)	Response Time (s)	Sensitivity (µA/cm^2^ × mg∙kg^−1^)	Reproducibility	Repeatability	Signal Recovery
Graphene-thiophene/Au	0.01 M TCA	100–200	13.8	46.06 ppm	551	17.82	0.49933	1.13–2.3% (*n* = 5)	1.5 (*n* = 3)	73.99–109%

**Table 2:** Performance comparison of the graphene-thiophene composite sensor for histamine detection with other reported sensors.

Sensors	Method	Concentration Range (mg/kg)	LOD (mg/kg)	[Ref]
AuNP/p-aminobenzene sulfonic acid/GCE	DPV	1420–3799	21.3	[3]
Fe_3_O_4_/Au@ATP@Ag	SERS	71–710	35.5	[20]
Au NPs-nitrilotriacetic acid-Ni^2+^ (NTANi^2+^)	SERS	35.5–3550	35.5	[21]
Fe_3_O_4_@SiO_2_–COOH -Ag NPs with glycine (Gly) and (3-Aminopheyonyl) boronic acid (APBA)	SERS	0.355–3550	0.257	[22]
Hydroxyl functionalized Schiff base zinc(II) complex/TiO_2_ NP/FTO	EIS	3.5–355	3.5	[29]
Ni@C metallic-organic framework/GCE	CV	3550–35,500	11,360	[30]
Graphene/MWCNT/AuNP	Transconductance	106.5–3550	3.55	[31]
Apt/AuNFs/ITO	DPV	0.03–177.5	0.02	[34]
Diamine oxidase phase-change microcapsules	CV	3550–284 × 10^3^	16.8	[35]
Graphene/antibodies with HRP-tagged histamine	Square Wave Voltammetry	0.00032–0.3195	0.00016	[40]
Fe_2_O_3_-TiO_2_-CdSe	Fluorescence	2485–159,750	56.8	[18]
Alizarin complexone (ALC) and Ni^2+^	UV-VIS	177.5–5325	272.64	[17]
Polystyrene–graphene oxide nanocomposite	DPV	3.55–106.5	1.065	[37]
Graphene nanoribbons-AgNPs	Square Wave Voltammetry	2130–17,750	0.6745	[38]
Our sensor (graphene-thiophene) in 0.01M histamine-TCA solution	CV	100–200	13.8	Our work

## Data Availability

Data are available upon request to the corresponding authors in a reasonable time frame.

## Supplementary Materials

The following supporting information can be downloaded at: https://www.mdpi.com/article/10.3390/foods12152908/s1. Figure S1: Cyclic voltammetry for the graphen-tiophene composite deposited onto Au support–20 cycles; Figure S2: Raman spectra of the sensitive layer of the polymerized graphene-thiophene deposited onto DRP-220AT; Figure S3: Cyclic voltammetry for different concentrations of histamine dispersed in phosphate buffer solution; Figure S4: Analysis of the concentration of histamine in PBS vs. (a) anodic/cathodic peak intensity and (b) charge of the anodic/cathodic peaks; Figure S5: Establishing the optimal concentration of the mediator: (a) CV of the different concentrations of TCA and (b) plot of the anodic peak intensity and charge for those concentrations; Figure S6: Variation of the square root of the scanning speed vs. anodic peak intensity for the histamine 200 ppm sample.
