# Supplementary material for: Spectro-Electrochemical Properties of A New Non-Enzymatic Modified Working Electrode Used for Histamine Assessment in the Diagnosis of Food Poisoning
Source: Foods. 2023 Jul 31;12(15):2908. doi: 10.3390/foods12152908 (PMC10417452; doi:10.3390/foods12152908)
Supplement: Supplementary file 1 [file foods-12-02908-s001.zip › foods-2437674-supplementary.pdf]

Supplementary File

Spectro-Electrochemical Properties of A New Non-Enzymatic Modified Working Electrode Used for Histamine Assessment in the Diagnosis of Food Poisoning

Stefan-Marian Iordache [1], Ana-Maria Iordache [1,*], Alexei Zubarev [2,*], Stefan Caramizoiu [3], Cristiana Eugenia Ana Grigorescu [1], Silviu Marinescu [4] and Carmen Giuglea [4]

1 Optospintronics Department, National Institute for Research and Development for Optoelectronics—INOE 2000, Atomistilor 409, 077125 Magurele, Romania; stefan.iordache@inoe.ro (S.-M.I.); krisis812@yahoo.co.uk (C.E.A.G.)
2 National Institute for Laser, Plasma and Radiation Physics, 077125 Magurele, Romania
3 National Institute for R&D in Microtechnologies IMT-Bucharest, 126A Erou Iancu Nicolae Str., 077190 Voluntari, Romania; stefancaramizoiu@yahoo.com
4 Department of Plastic Surgery, University of Medicine and Pharmacy "Carol Davila", Eroii Sanitari Bvd., No. 8, Sector 5, 050471 Bucharest, Romania; silviu.marinescu@umfcd.ro (S.M.); giugleacarmen@yahoo.com (C.G.)
* Correspondence: ana.iordache@inoe.ro (A.-M.I.); alxzubarev@gmail.com (A.Z.)

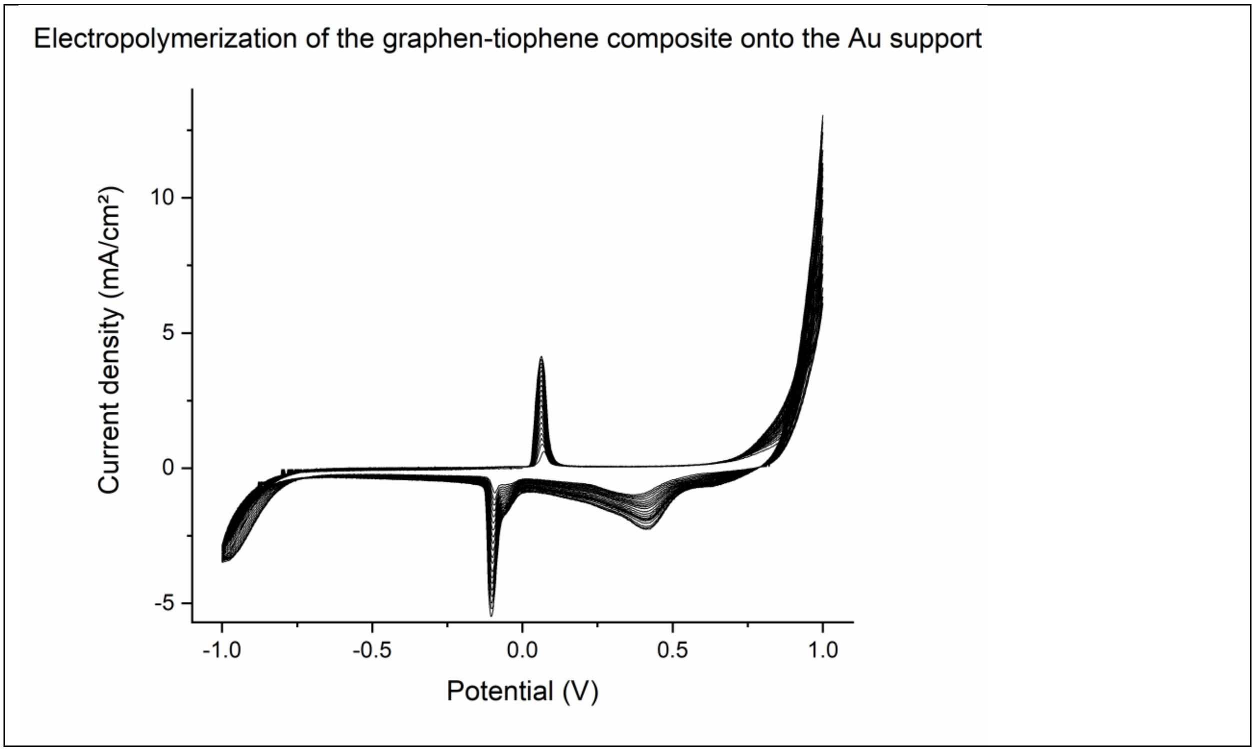

Figure S1. Cyclic voltammetry for the graphen-tiophene composite deposited onto Au support – 20 cycles.

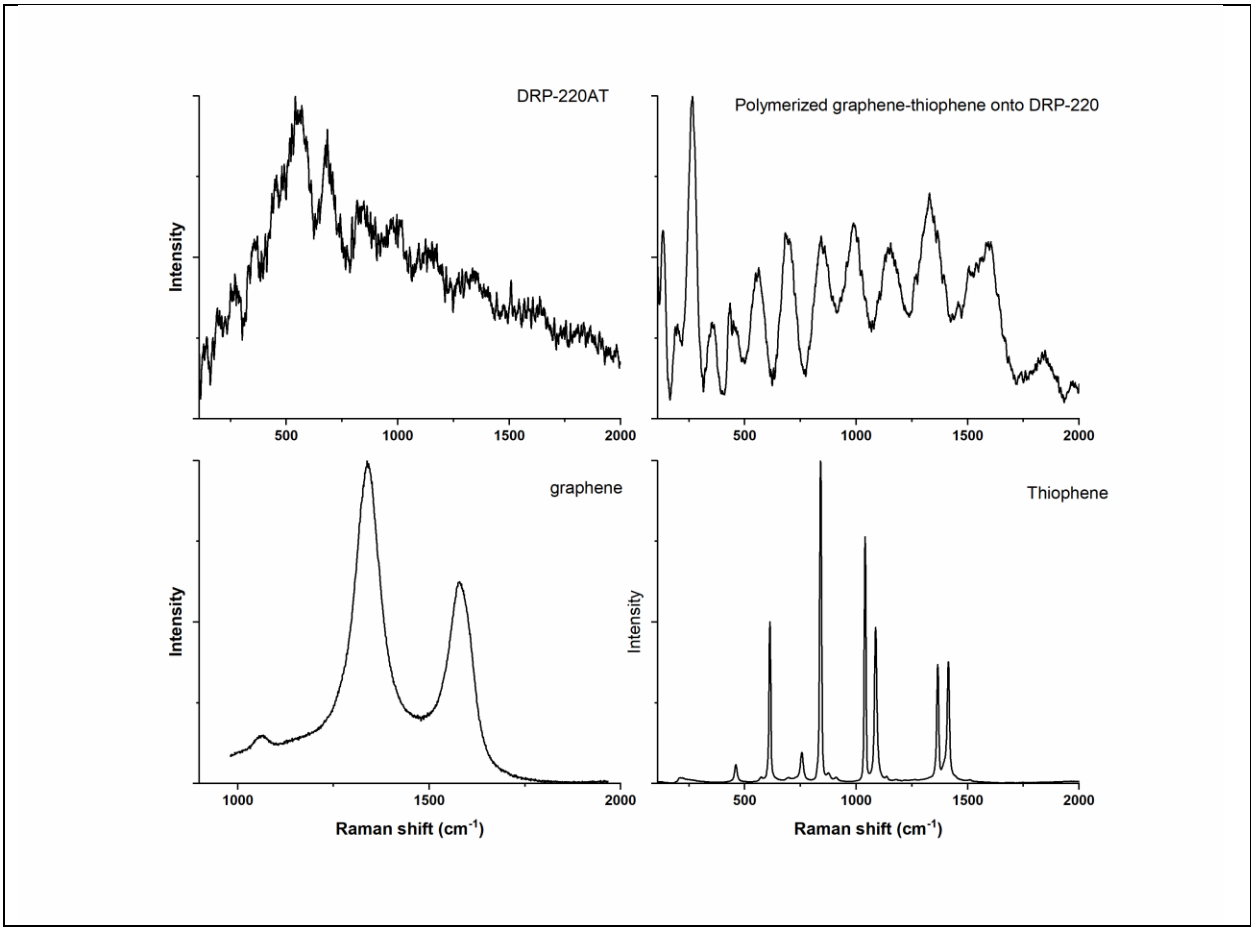

Figure S2. Raman spectra of the sensitive layer of the polymerized graphene-thiophene deposited onto DRP-220AT.

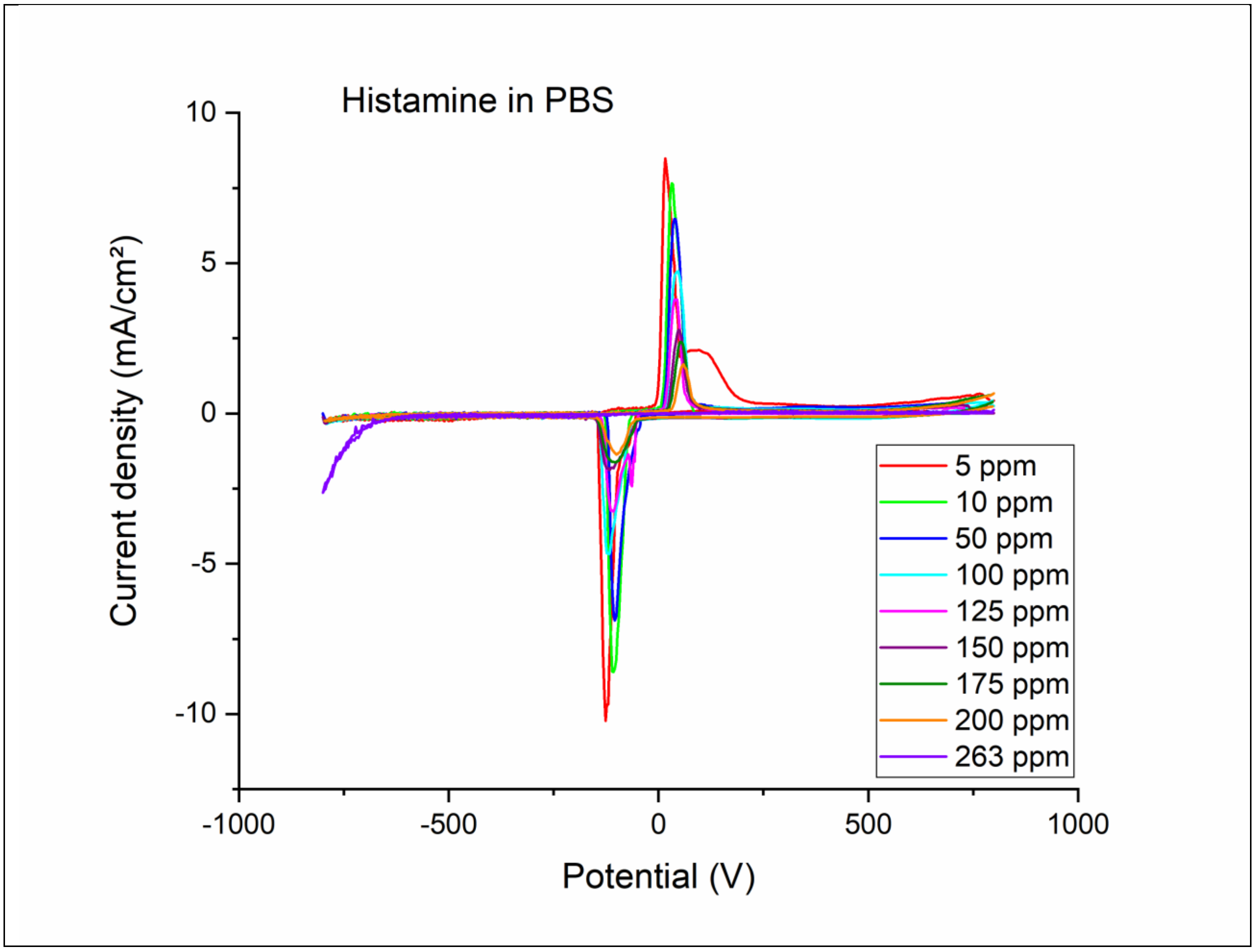

Figure S3. Cyclic voltammetry for different concentrations of histamine dispersed in phosphate buffer solution.

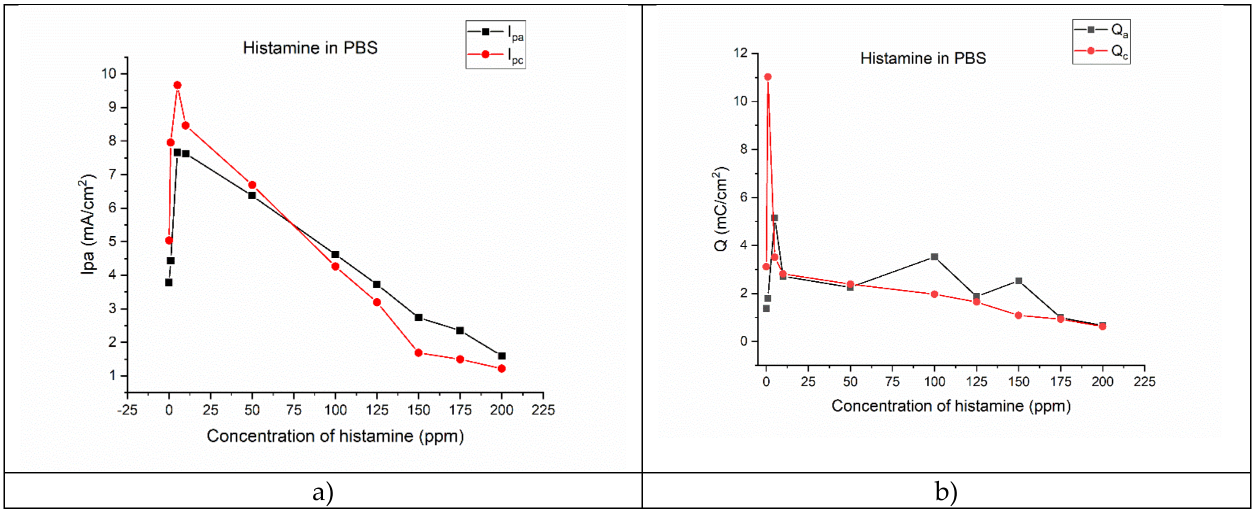

Figure S4. Analysis of the concentration of histamine in PBS *vs.* a) anodic/cathodic peak intensity and b) charge of the anodic/cathodic peaks.

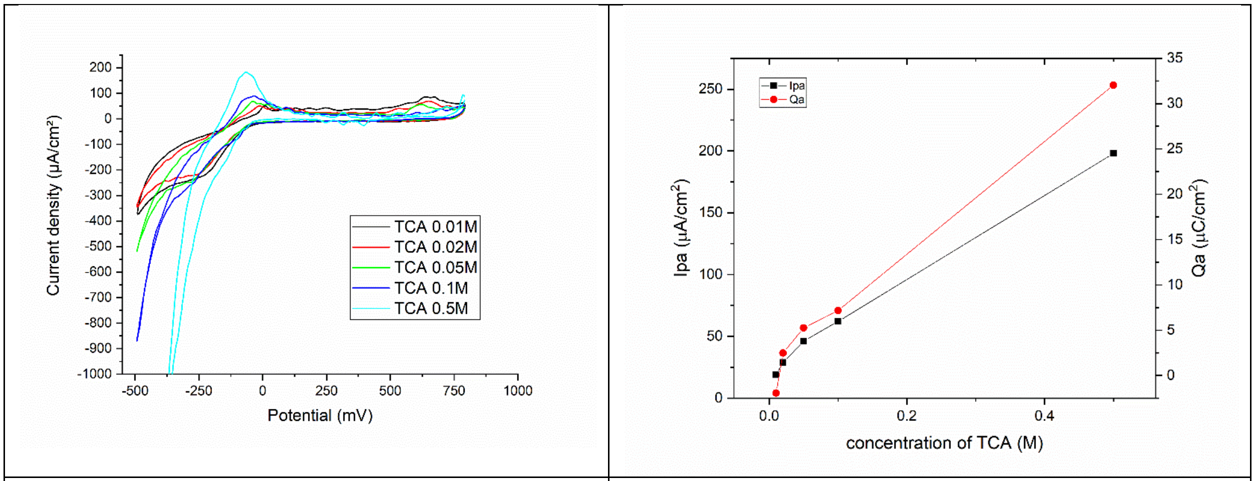

a)	b)

Figure S5. Establishing the optimal concentration of the mediator: a) CV of the different concentrations of TCA and b) plot of the anodic peak intensity and charge for those concentrations.

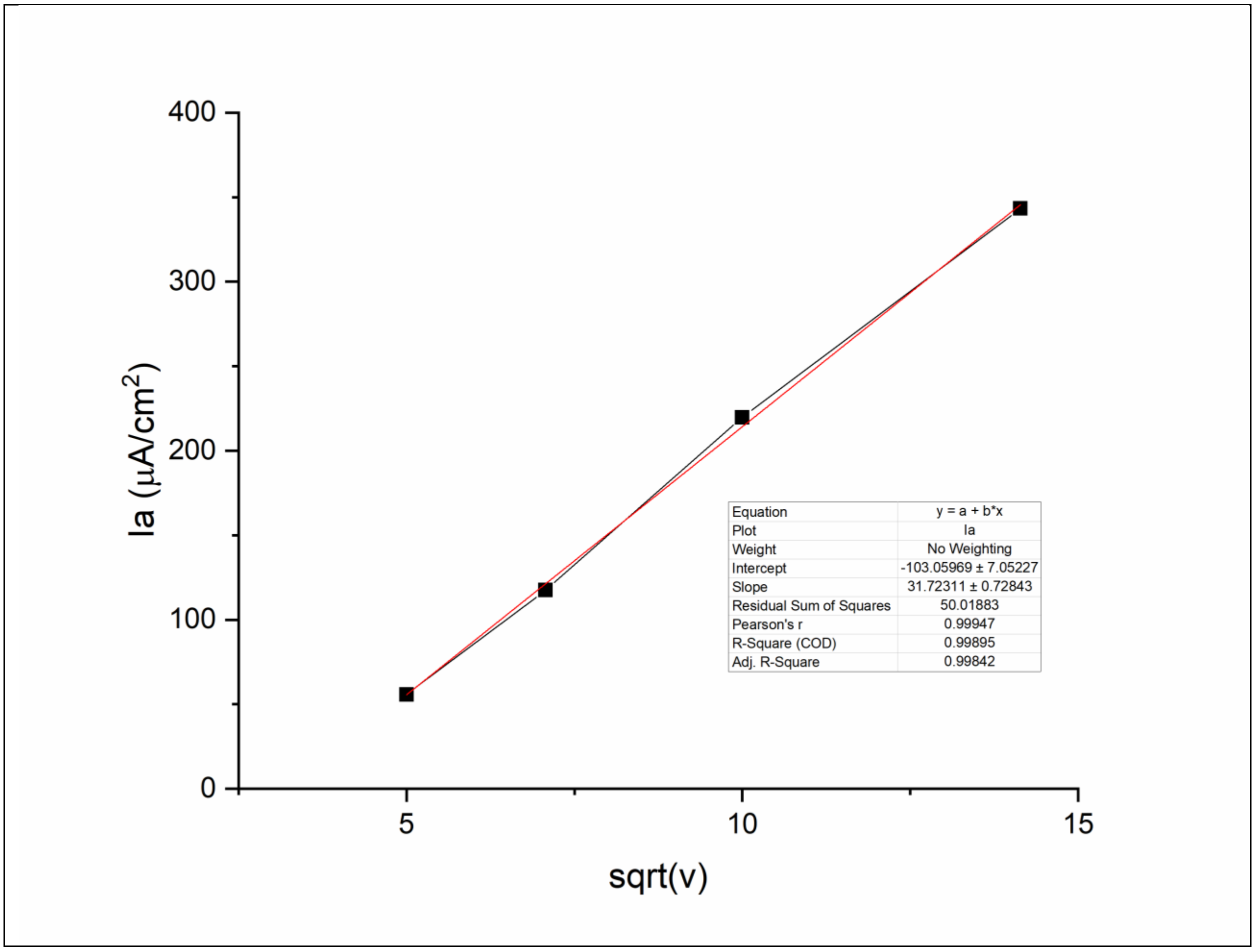

Figure S6. Variation of the square root of the scanning speed vs. anodic peak intensity for the histamine 200 ppm sample.

Formulas for calculating the analytical parameters:

1. $\%RSD = \frac{SD*100}{mean}$, where SD is the standard deviation, calculated as 5.2 for the 5 cycles of the cyclic voltammetry
2. $LOD = \frac{3*\%RSD}{slope}$, %RSD is the relative standard deviation and the slope is given by the linear equation of the Ipa vs concentration
3. $LOQ = \frac{10*\%RSD}{slope}$
4. $reproducibility = \%RSD$
5. $repeatability = \frac{SD}{\sqrt{number\ of\ samples}}$, SD = 2.6 (testing the same concentration on 3 microelectrodes), no of samples=3
6. $Sensibility = slope\ of\ the\ Ipa\ vs\ concentration = 0.49933\ \frac{\mu A}{cm^2*ppm}$
